# Development of bright red‐shifted miRFP704nano using structural analysis of miRFPnano proteins

**DOI:** 10.1002/pro.4709

**Published:** 2023-08-01

**Authors:** Olena S. Oliinyk, Sergei Pletnev, Mikhail Baloban, Vladislav V. Verkhusha

**Affiliations:** ^1^ Medicum, Faculty of Medicine University of Helsinki Helsinki Finland; ^2^ Vaccine Research Center, National Institute of Allergy and Infectious Diseases National Institutes of Health Bethesda Maryland USA; ^3^ Department of Genetics and Gruss‐Lipper Biophotonics Center Albert Einstein College of Medicine Bronx New York USA

**Keywords:** BphP, CBCR, iRFP, miRFP670nano, NIR FP, phytochrome

## Abstract

We recently converted the GAF domain of NpR3784 cyanobacteriochrome into near‐infrared (NIR) fluorescent proteins (FPs). Unlike cyanobacterichrome, which incorporates phycocyanobilin tetrapyrrole, engineered NIR FPs bind biliverdin abundant in mammalian cells, thus being the smallest scaffold for it. Here, we determined the crystal structure of the brightest blue‐shifted protein of the series, miRFP670nano3, at 1.8 Å resolution, characterized its chromophore environment and explained the molecular basis of its spectral properties. Using the determined structure, we have rationally designed a red‐shifted NIR FP, termed miRFP704nano, with excitation at 680 nm and emission at 704 nm. miRFP704nano exhibits a small size of 17 kDa, enhanced molecular brightness, photostability and pH‐stability. miRFP704nano performs well in various protein fusions in live mammalian cells and should become a versatile genetically‐encoded NIR probe for multiplexed imaging across spatial scales in different modalities.

## 1 INTRODUCTION

Modern biomedicine is increasingly reliant on non‐invasive near‐infrared (NIR) optical imaging. However, the limitation in this field is the availability of genetically encoded probes, such as NIR fluorescent proteins (FPs), to study processes deeply in organisms in vivo.

Most of the NIR FPs were engineered from bacterial phytochrome photoreceptors (BphPs) that utilize as a chromophore biliverdin IXα (BV), available in mammalian cells (Tran et al., 2014; Shcherbakova et al., 2015). For example, spectrally distinct BphP‐based NIR FPs of the miRFP series, including miRFP670, miRFP703 and miRFP709, were engineered to specifically incorporate endogenous BV in mammalian cells (Shcherbakova et al., 2016; Shemetov et al., 2017; Oliinyk et al., 2017). BphP‐based FPs consist of PAS and GAF domains interlinked by a figure‐of‐eight knot structure (Wagner et al., 2005) and have a relatively high molecular weight of 35 kDa. Although BV resides in the GAF domain of BphP‐derived FPs, both PAS and GAF domains are required for chromophore binding. We reported miRFP670nano protein, the first single‐domain NIR FP developed from a GAF domain of cyanobacteriochrome (CBCR) photoreceptor, which only requires GAF domain to covalently attach chromophore (Oliinyk et al., 2019).

By applying rational design and directed molecular evolution to miRFP670nano, we have recently developed miRFP670nano3 and miRFP718nano, its new mutants with unique features (Oliinyk et al., 2022, 2023).

The main advantage of miRFP670nano3 is its substantially improved brightness. miRFP670nano3 has spectral properties similar to those of parental miRFP670nano, but its molecular brightness (the product of a molar extinction coefficient and a quantum yield) is more than twofold higher than the brightness of miRFP670nano (Table 1) (Oliinyk et al., 2022). Importantly, the fluorescence quantum yield of miRFP670nano3 almost twice outperforms the quantum yield of its progenitor and is the highest among all monomeric NIR FPs with BV chromophore (Table 1) (Oliinyk et al., 2022). Interestingly, in mammalian cells the brightness of miRFP670nano3 fourfold outperforms the brightness of miRFP670nano (Table 1) (Oliinyk et al., 2022).

**TABLE 1:** Major spectral and biochemical properties of miRFPnano proteins and selected two‐domain BphP‐based proteins.

NIR FP	Ex (nm)	Em (nm)	Extinction coefficient (M^−1^ cm^−1^)	Quantum yield (%)	Molecular brightness vs. miRFP670nano (%)	p*K*a_1_ (acid)	p*K*a_2_ (alkaline)	Photostability in HeLa cells, *t*_1/2_ (s)	Brightness in mammalian cells vs. miRFP670nano (%)	Ref.
miRFP670nano	645	670	95,000	10.8	100	3.7	8.0	545	100	Oliinyk et al. (2019)
miRFP670nano3	645	670	129,000	18.5	233	4.2	11	675	412	Oliinyk et al. (2022)
**miRFP704nano**	**680**	**704**	**93,000**	**9.9**	**90**	**4.1**	**>11**	**1265**	**134**	**This paper**
miRFP718nano	690	718	79,000	5.6	43	3.8	10.5	885	55	Oliinyk et al. (2023)
mIFP	683	704	82,000	8.4	67	4.5	9.2	54	26	Yu et al. (2015) and Shemetov et al. (2017)
miRFP703	674	703	90,900	8.6	76	4.5	>9.5	394	61	Shcherbakova et al. (2016)
miRFP709	683	709	78,400	5.4	41	4.5	9.2	192	42	Shcherbakova et al. (2016)
miRFP720	702	720	98,000	6.1	58	4.5	>9.5	510	145	Shcherbakova et al. (2018)

miRFP718nano is substantially red‐shifted compared to parental miRFP670nano. It has an excitation band maximum at 690 nm, which is 50 nm red‐shifted compared to an excitation maximum of miRFP670nano at 640 nm. The emission spectrum of miRFP718nano is characterized by a maximum at 718 nm, which is 48 nm red‐shifted compared to miRFP670nano, having an emission maximum at 670 nm (Table 1) (Oliinyk et al., 2023). Redshift in miRFP718nano was accompanied by a decrease in quantum yield, and its molecular and cellular brightness are twice lower than those of miRFP670nano (Table 1). Although the brightness of miRFP718nano is compatible with the brightness of spectrally close NIR FPs, miRFP718nano is substantially dimmer than parental miRFP670nano. We, therefore, embarked on the project to generate brighter red‐shifted NIR FP.

In this work, we first report on the crystal structure of miRFP670nano3 to explain the effect of the introduced mutations on the spectral properties of the miRFPnano family. We then engineer and characterize in vitro and mammalian cells a miRFP704nano NIR FP with enhanced brightness.

## 2 RESULTS

### 2.1 Structural basis for the properties of miRFP670nano3

To engineer bright red‐shifted single‐domain NIR FP, we combined properties of red‐shifted miRFP718nano and bright miRFP670nano3 mutants. For this, we determined the crystal structure of miRFP670nano3 and used analysis of miRFP718nano and miRFP670nano3 structures to rationally engineer enhanced red‐shifted NIR FP.

**FIGURE 1:**
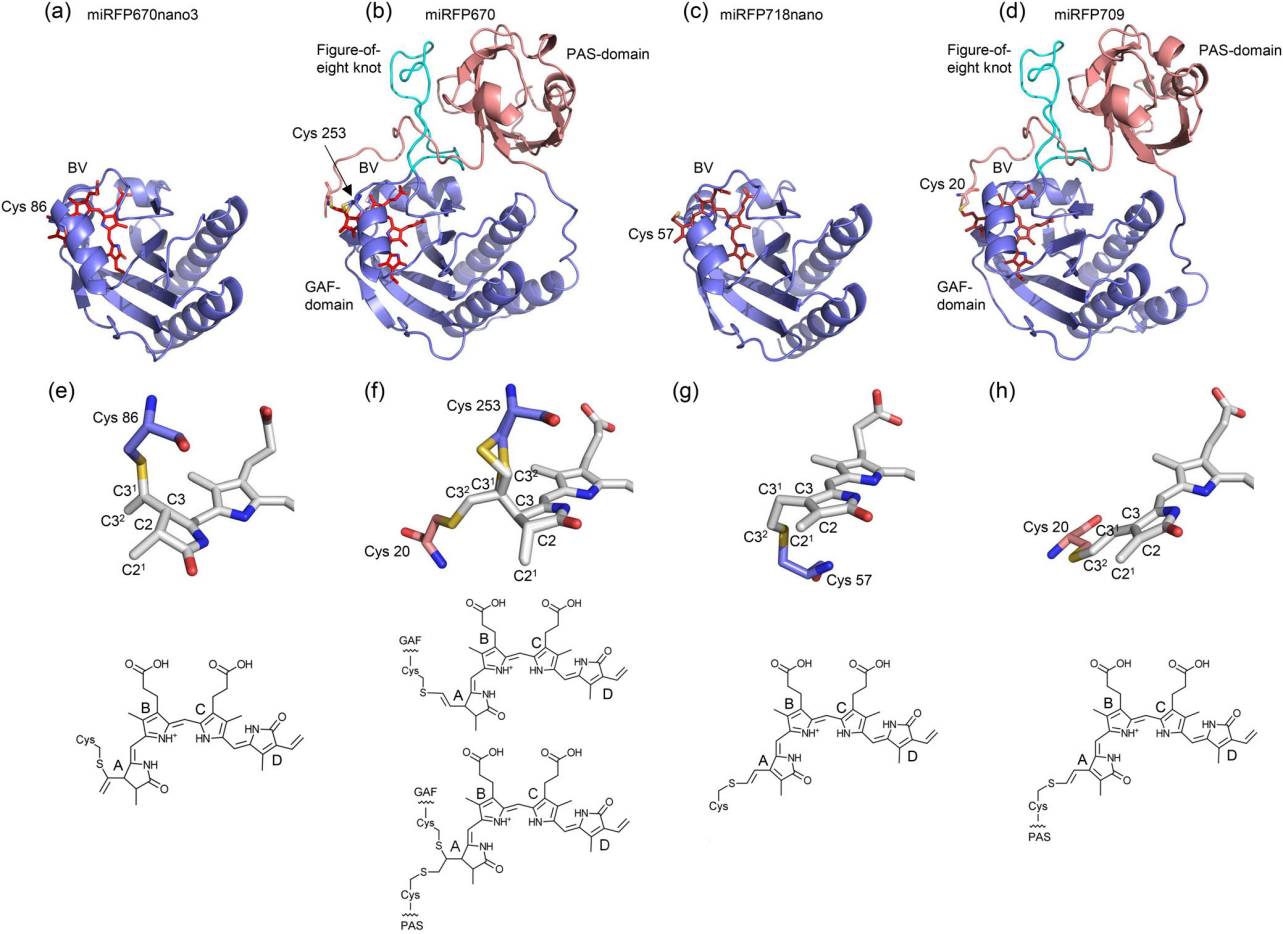
Comparison of two‐domain miRFPs and single‐domain miRFPnanos structures and their chromophores. Overall structures of (a) miRFP670nano3, (b) miRFP670 (PDB ID: 5VIV), (c) miRFP718nano (PDB ID: 7LSD), and (d) miRFP709 (PDB ID: 5VIQ). The biliverdin (BV) chromophores are in red. In miRFPnanos, the α1‐helix is removed. The PAS and GAF domains of miRFPs are in pink and blue, respectively. The BV chromophores in (e) miRFP670nano3, (f) miRFP670, (g) miRFP718nano, and (h) miRFP709 bound to the respective Cys residues and their chemical formulas. Carbon, nitrogen, oxygen, and sulfur atoms are in white, blue, red, and yellow, respectively. Sticks representations show only rings A and B of the chromophores and Cys residues. In miRFP670nano3, the BV chromophore (e) is bound to Cys86 via the C3^1^ atom. In miRFP670 (f), two chromophore‐binding modes are observed. In this protein, the BV can be bound to either Cys253 in the GAF domain via its C3^2^ atom (upper formula), or to both Cys253 in the GAF, and Cys20 in the PAS domain via its C3^1^ and C3^2^ atoms, respectively (lower formula). miRFP718nano (g) and miRFP709 (h) have the same chromophore species bound to the Cys57 and Cys20, respectively.

**FIGURE 2:**
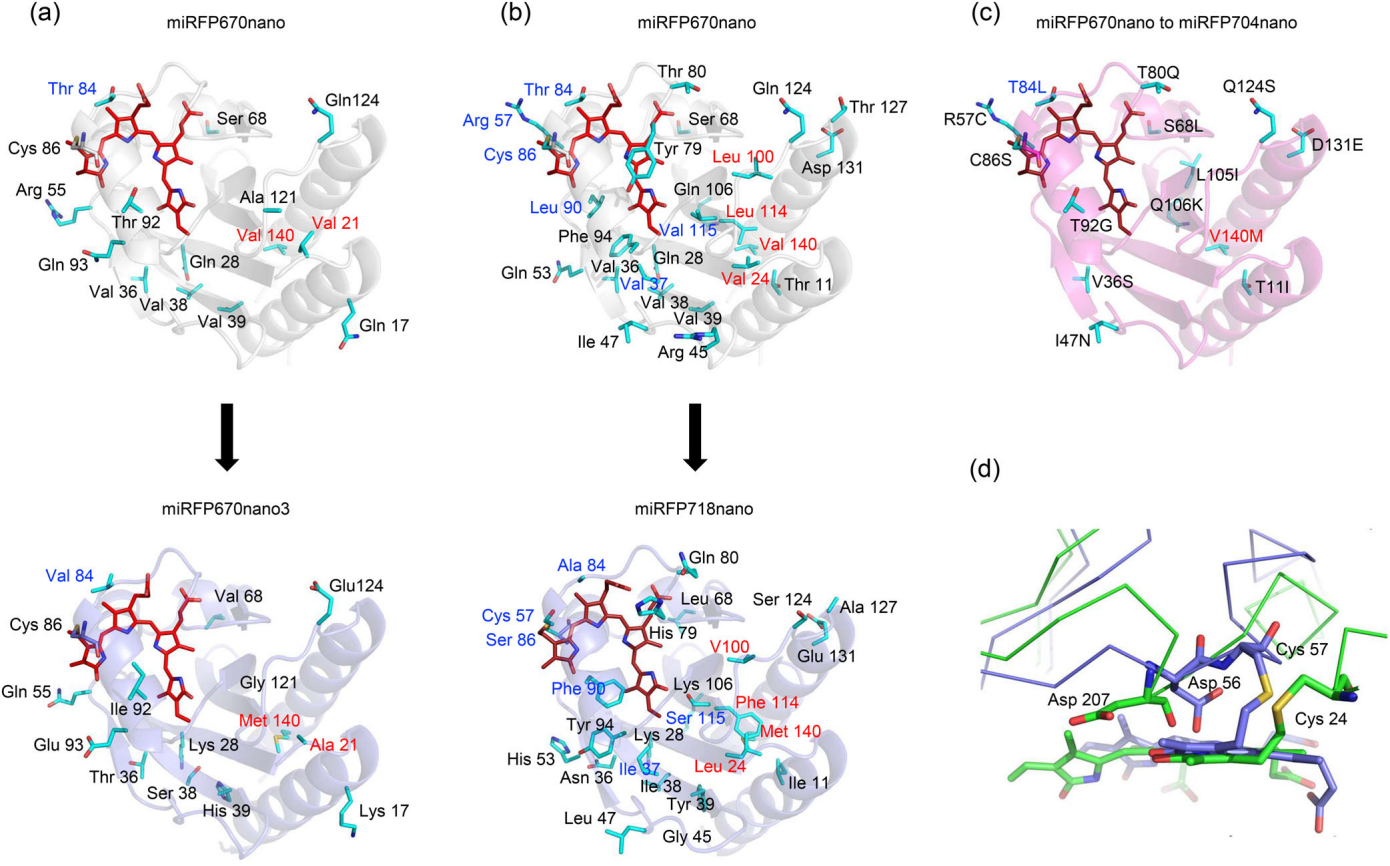
Differences in amino acid residue composition between parental and progenitor miRFPnanos. (a) The difference between miRFP670nano and miRFP670nano3. (b) The difference between miRFP670nano and miRFP718nano. (c) The difference between miRFP670nano and miRFP704nano. Surface residues are black, buried residues are red, and the residues contacting BV are blue. (d) The difference in positions of Asp207 in phytochromes and Asp56 in cyanobacteriochromes. The chromophore‐binding domain of *Deinococcus radiodurans* phytochrome (PDB ID: 1ZTU) is green and miRFP718nano (PDB ID: 7LSD) is slate.

**FIGURE 3:**
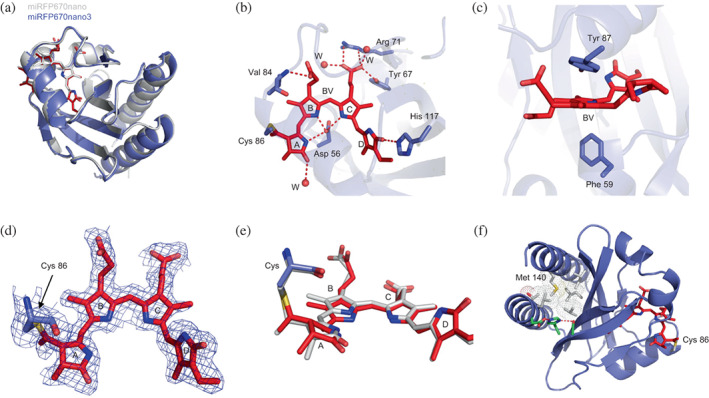
Structure of miRFP670nano3 and the environment of its chromophore. (a) Superposition of miRFP670nano (PDB ID: 5VIV) (white) and miRFP670nano3 (blue) structures. (b) Hydrogen bond network around the chromophore in miRFP670nano3. (c) Stacking interactions between the chromophore and the surrounding residues in miRFP670nano3. (d) The chromophore of miRFP670nano3 bound to Cys86 residue in *2Fo‐Fc* electron density map countered at 1.0 σ level. (e) Superposition of the chromophores in miRFP670nano (white) and miRFP670nano3 (red). (f) Hydrophobic cluster in miRFP670nano3 (shown as white spheres).

The crystal structure of miRFP670nano3 has been solved to a resolution of 1.8 Å. We compared it with parental miRFP670nano (Oliinyk et al., 2019), with which it shares ~90% of the sequence identity (Figures 1, 2, 3). The RMS deviation between miRFP670nano and miRFP670nano3 superimposed by C^α^ atoms does not exceed 0.74 Å. The proteins have a similar GAF fold with the N‐ and C‐termini positioned close to each other. This fold is similar to that of the GAF domains of BphP‐based NIR FPs, miRFP670 and miRFP709, linked to the adjacent PAS domain (Figure 1b,d) (Baloban et al., 2017) that is absent in miRFPnanos derived from a single GAF‐domain only CBCR (Oliinyk et al., 2019, 2023).

miRFP670nano3 differs from its progenitor miRFP670nano by 14 amino acid residues, 12 of which are located on the surface and two buried inside the protein (Figure 2a) (Oliinyk et al., 2022). In both miRFP670nano and miRFP670nano3, the chromophore forms a covalent thioether bond between the C3^1^ atom of ring A of BV and cysteine residue conserved in CBCRs (C86 residue in miRFP670nano and miRFP670nano3) (Figures 1e and 3d,e). The formation of the covalent bond is accompanied by a reduction of ring A, shortening of the conjugated system of the chromophore and hence a hypsochromic shift of its fluorescence. This chromophore attachment is different from that found in BphP‐based NIR FPs miRFP703, miRFP709, and miRFP670. In red‐shifted miRFP703 and miRFP709, BV is attached to the protein by the C3^2^ atom of ring A, forming a covalent bond with conserved C20 residue in the PAS domain and ring A remains oxidized (Figure 1h) (Baloban et al., 2017). In blue‐shifted miRFP670, BV attachment, accompanied by ring A reduction, is realized either as a single covalent bond between C3^2^ atom of ring A and C253 residue in the GAF domain or as two covalent bonds between C3^1^ atom of ring A and C253 residue in the GAF domain and C3^2^ atom and C20 residue in the PAS domain (Figure 1f) (Baloban et al., 2017). In miRFP670nano and miRFP670nano3, BV chromophore has the same number of conjugated bonds as in miRFP670 reflected in the similarity of their fluorescence spectra.

### 2.2 Chromophore environment in miRFP670nano3

The immediate chromophore environment in miRFP670nano and miRFP670nano3 is rather similar. The chromophore is stabilized by eight hydrogen bonds with the nearby residues D56, Y67, R71, V84, and H117, three hydrogen bonds with water molecules, a face‐to‐face stacking with Y87, and an edge‐to‐face stacking with F59 (Figure 3b,c). H117 forms a stabilizing hydrogen bond with ring D of BV, preventing its rotation and photoisomerization. The positions of propionate groups of rings B and C are stabilized by H‐bonds with the main chain nitrogen of V84 and the side chains of R71 and Y67, respectively. Ring A carbonyl and ring C propionate form H‐bonds with three water molecules. Finally, the middle part of the chromophore (parts of rings B and C and the methine bridge between them) is sandwiched between residues Y87 and F59, presumably providing for high extinction coefficients and hence the brightness of these proteins.

Substantially increased QY and brightness of miRFP670nano3 are likely provided by three key mutations V38S, V39H, and V140M. The neighbor V38S and V39H mutations act cooperatively: the side chain of S38 forms a strong H‐bond with the side chain of H39 locking it in the conformation favoring H‐bonding with the side chain of D5. This H‐bond stabilizes the spatial position of the N‐terminal α‐helix, tethering it to the second β‐strand of the protein. The long side chain of M140 is the central element of the dense hydrophobic cluster formed by residues L8, T11, V26, I104, L114, and M140 (Figure 3f). This cluster effectively “glues” N‐ and C‐terminal α‐helices to the central β‐sheet of the protein, making the fold of miRFP670nano3 more rigid than in parental miRFP670nano. The enhanced rigidity of the protein restricts BV mobility, makes the non‐radiative transition of the chromophore less favorable, and hence provides for increased QY and brightness of miRFP670nano3. The introduction of hydrophobic V84 next to the BV could also be accountable for the increased quantum yield of miRFP670nano3. Two other mutations, V21A and A121G, removed steric tension with nearby R123 and P122, respectively, presumably improving protein fold.

### 2.3 Structural basis for the properties of red‐shifted miRFPnanos

**FIGURE 4:**
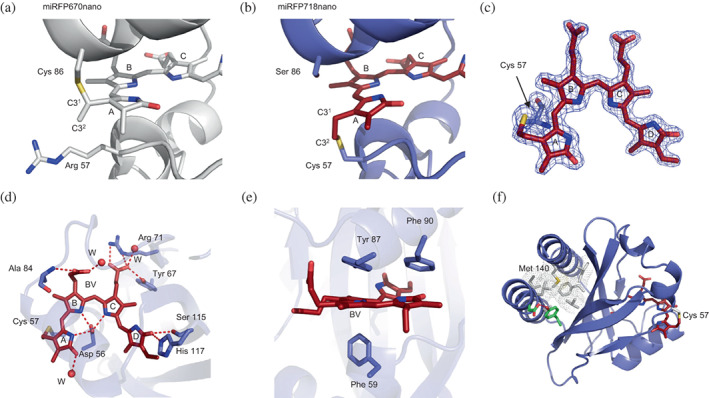
Structure of miRFP718nano and the environment of its chromophore. (a) The structure of miRFP670nano (PDB ID: 5VIV) reveals a close location of Arg57 to the C3^2^ atom of the chromophore. (b) The structure of miRFP718nano (PDB ID: 7LSD). The chromophore is colored dark red. (c) The chromophore of miRFP718nano bound to Cys57 residue in *2Fo‐Fc* electron density map countered at 2.0 σ level. (d) Hydrogen bond network around the chromophore in miRFP718nano. (e) Stacking interactions between the chromophore and the surrounding residues in miRFP718nano. (f) Hydrophobic cluster in miRFP718nano (shown as white spheres).

The crystal structure of miRFP670nano reported earlier revealed that C^β^ and C^γ^ atoms of R57 are positioned close to the C3^2^ atom of ring A of BV (Oliinyk et al., 2019) (Figure 4a). Wagner et al. noted that the formation of the thioether bond requires the presence of a base (Wagner et al., 2005) to activate the cysteine thiol for subsequent nucleophilic attack at C3^2^ atom of BV. In miRFP670nano the carboxyl of E61 is within 3.5 Å, from the C^β^ of R57 fulfilling this requirement. We hypothesized that coupling substitutions R57C and C86S could enable a covalent binding of BV by the C3^2^ atom. The reduction of BV ring A would not accompany such binding, providing for red‐shifted emission of the chromophore. Indeed, the first variant miRFP670nano/R57C/C86S emitted light at 705 nm, demonstrating the correctness of this structure‐based supposition. Random mutagenesis of miRFP670nano/R57C/C86S resulted in the new protein miRFP718nano. We obtained the crystals for miRFP718nano and reported its structure previously (Oliinyk et al., 2023). miRFP718nano differs from its progenitor miRFP670nano by 26 amino acid residues, 21 of which are located on the surface and 5 buried inside the protein (Figure 2b) (Oliinyk et al., 2023). Residues, I37, C57, A84, S86, F90, and S115 are part of the immediate environment of the chromophore. miRFP718nano and BphP‐based NIR FP miRFP709 (PDB ID: 5VIQ) have the same chromophore that forms a covalent thioether bond between the C3^2^ atom of BV ring A and the rationally introduced C57 residue or the conserved C20 residue of the PAS domain, respectively (Figures 1g,h and 4b,c). Compared with miRFP670nano3, miRFP718nano has an extra H‐bond between ring D of BV and S115 (V115 in miRFP670nano3) and the third tilted face‐to‐edge stacking between ring D and F90 (L90 in miRFP670nano3) (Figure 4d,e). This H‐bond and stacking interaction provide an additional bathochromic shift of miRFP718nano emission (Oliinyk et al., 2023). Same as in miRFP670nano3, miRFP718nano contains a massive hydrophobic cluster between terminal α‐helices and internal β‐sheet enhancing protein stability (Figure 4f).

### 2.4 Rational design and molecular evolution of miRFP704nano

**FIGURE 5:**
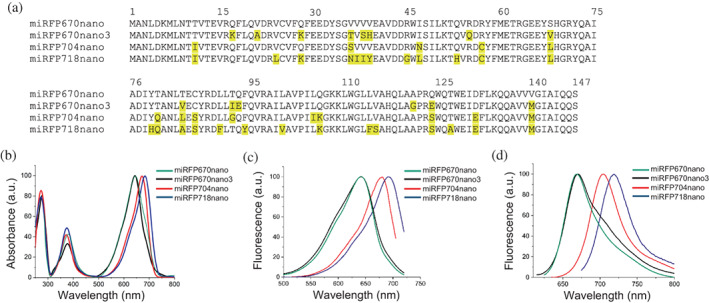
Sequences and spectral properties of miRFP704nano and other members of miRFP670nano family, such as miRFP670nano, miRFP670nano3, and miRFP718nano. (a) Alignment of the amino acid sequences of miRFPnanos. (b) Absorption spectra of the proteins. (c) Fluorescence excitation spectra of miRFPnanos (emission at 730 nm for miRFP670nano, miRFP670nano3, miRFP704nano and 740 nm for miRFP718nano, respectively). (d) Fluorescence emission spectra of miRFPnanos (excitation at 600 nm for miRFP670nano and miRFP670nano3, 620 nm for miRFP704nano and 650 nm for miRFP718nano, respectively).

To engineer bright red‐shifted single‐domain NIR FP, we relied on analysis of the structures of bright miRFP670nano3 mutant and red‐shifted miRFP718nano mutant. As a starting point, we used miRFP670nano/R57C/C86S double mutant, whose fluorescence spectra are 35 nm red‐shifted as compared to parental miRFP670nano. miRFP670nano/R57C/C86S was subjected to saturated mutagenesis at positions identified during directed molecular evolution of miRFP670nano3 and miRFP718nano (Figure 5a). On each round of molecular evolution, bacterial libraries of mutants were first screened with the fluorescence‐activated cell sorting (FACS), using a 640 nm excitation laser and a 725/40 nm emission filter. The brightest collected bacterial cells were then plated on Petri dishes and screened again with the fluorescence stereomicroscope using a 700/20 nm excitation and a 730 nm long‐pass emission filters for positive selection and a 615/30 nm excitation and a 670/30 nm emission filters to exclude blue‐shifted mutants. After initial screening, the fluorescence spectra of selected clones were additionally analyzed by fluorimeter and blue‐shifted mutants were discarded. The fluorescence intensity of 20–30 brightest red‐shifted clones was analyzed in transiently transfected HeLa cells with no supply of exogenous BV. A mixture of the selected in mammalian cells mutants was then used as a template for the next round of mutagenesis. As a result, we obtained NIR FP, named miRFP704nano, having 14 mutations relative to parental miRFP670nano (Figures 2c and 5a).

### 2.5 Spectral properties of miRFP704nano protein

The absorbance spectrum of miRFP704nano was characterized by a major absorbance peak at 680 nm, whereas miRFP670nano and miRFP670nano3 had an absorbance maximum at 645 nm, and miRFP718nano had an absorbance maximum at 688 nm. The absorbance spectra of all NIR FPs contained a minor peak at 390 nm, corresponding to the Soret band, which is characteristic of tetrapyrrole‐binding proteins (Figure 5b and Table 1). miRFP704nano had an excitation maximum at 680 nm, which was 40 nm red‐shifted compared with excitation peaks of miRFP670nano and miRFP670nano3 at 640 nm (Figure 5c and Table 1). The emission spectrum of miRFP704nano was characterized by a maximum at 704 nm, which was 34 nm red‐shifted, compared with miRFP670nano and miRFP670nano3, having the emission maximums at 670 nm (Figure 5d and Table 1). Thus, miRFP704nano is substantially red‐shifted compared with miRFP670nano and miRFP670nano3 FPs but is slightly blue‐shifted compared with miRFP718nano, having excitation/emission maxima at 690/718 nm (Figure 5c,d and Table 1).

### 2.6 Biochemical properties of miRFP704nano protein

The molecular brightness of miRFP704nano was twice higher than that of miRFP718nano and close to the brightness of miRFP670nano. Importantly, both, the extinction coefficient (93,000 M^−1^ cm^−1^) and fluorescence quantum yield (9.9%) of miRFP704nano were larger than those of the spectrally close two‐domain NIR FPs derived from BphPs, such as miRFP703 (Shcherbakova et al., 2016), miRFP709 (Shcherbakova et al., 2016) and mIFP (Yu et al., 2015; Shemetov et al., 2017) (Table 1).

**FIGURE 6:**
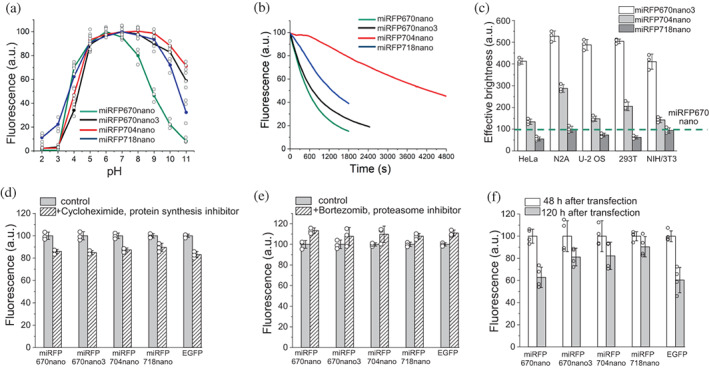
Biochemical and cellular properties of miRFP704nano in comparison with other miRFPnanos. (a) pH dependencies of NIR fluorescence. Data are presented as mean values ± SD for *n* = 3 experiments. (b) Photobleaching in live HeLa cells with 665/45 nm excitation filter. (c) Cellular brightness in live mammalian cells. Fluorescence was analyzed 72 h after transfection. The effective brightness of miRFP670nano was assumed to be 100% for each cell type. NIR fluorescence intensity was normalized to the excitation efficiency of each NIR FP by 640 nm laser and the emission spectrum of each FP in the emission filter. (d) Mean fluorescence intensity of HeLa cells transiently transfected with miRFPnanos and EGFP before and after 4 h of incubation with a protein synthesis inhibitor cycloheximide (20 μg mL^−1^). Fluorescence was normalized to control conditions. (e) Mean fluorescence intensity of HeLa cells transiently transfected with miRFPnanos and EGFP before and after 4 h of incubation with an inhibitor of proteasome‐dependent protein degradation bortezomib (10 μM). Fluorescence was normalized to control conditions. In (d, e) data are presented as mean values ± SD for *n* = 3 transfection experiments. (f) Mean fluorescence intensity of live HeLa cells transiently transfected with miRFPnanos and EGFP 48 and 120 h after transfection normalized to that at 48 h. Data are presented as mean values ± SD for *n* = 4 transfection experiments.

The fluorescence of miRFP704nano was stable over a wide pH range of 4.0–11.0. Compared to other miRFPnanos, miRFP704nano demonstrated improved resistance to alkaline environments (Figure 6a and Table 1).

miRFP704nano exhibited substantially improved photostability, which was 2.3‐fold higher than that of parental miRFP670nano (Figure 6b and Table 1). Judging from the photobleaching curve, we hypothesize that miRFP704nano exhibits a weak photochromicity (Figure 6b). The photostability of miRFP704nano was 3.3‐ and 23.4‐fold higher than that of spectrally close BphP‐based NIR FPs miRFP703 and mIFP, respectively (Table 1). High photostability in combination with high brightness makes miRFP704nano a favorable NIR‐FP for monitoring long‐term events.

### 2.7 Performance of miRFP704nano in live mammalian cells

Since the fluorescence of NIR FPs is provided by a BV chromophore, their brightness in mammalian cells (termed cellular or effective brightness) depends not only on molecular brightness but also on the efficiency of the binding of endogenous BV (Shemetov et al., 2017). To study an effective brightness of miRFP704nano, we transiently transfected HeLa, N2a, U‐2 OS, HEK293T, and NIH/3T3 cells with miRFP670nano‐, miRFP718nano‐, and miRFP704nano‐encoding plasmids and analyzed the NIR fluorescence of transfected cells by flow cytometry, using a 640 nm excitation laser and a 670 nm long‐pass emission filter. miRFP704nano was brightly fluorescent in all mammalian cell lines without adding exogenous BV. Although less bright than miRFP670nano3, miRFP704nano outperformed parental miRFP670nano and was more than twofold brighter than miRFP718nano (Figure 6c and Table 1 **).**

Intracellular protein stability assay showed that after incubation with a protein synthesis inhibitor cycloheximide, miRFP704nano‐expressing cells retained ~90% of their fluorescence intensity, demonstrating slightly higher protein stability than miRFP670nano‐ and EGFP‐expressing cells, which retained ~85% of their fluorescence (Figure 6d). To study degradation rate, cells expressing FPs were treated with bortezomib, an inhibitor of proteasome‐dependent protein degradation. Incubation with bortezomib increased the brightness of the miRFP704nano‐expressing cells only by 7%. Similarly, bortezomib treatment only slightly increased the brightness of cells, expressing other FPs (Figure 6e). We next analyzed the brightness of fluorescent cells at 48 and 120 h after transient transfection with miRFP704nano‐, miRFP718nano‐, or miRFP670nano‐ and EGFP‐encoding plasmids. The miRFP704nano‐expressing cells retained ~80% of their fluorescence intensity, whereas the miRFP670nano‐ and EGFP‐expressing cells only ~60% (Figure 6f). Taken together, these data demonstrated that miRFP704nano has high intracellular protein stability and exhibits bright fluorescence in different mammalian cell lines.

**FIGURE 7:**
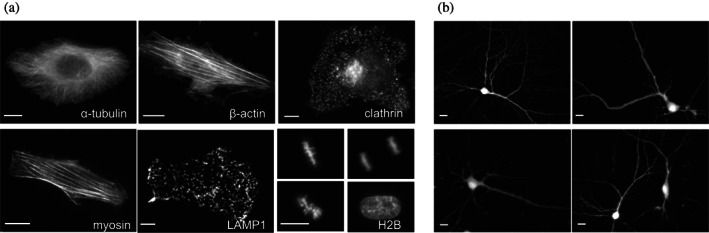
Protein fusions of miRFP704nano imaged using epifluorescence microscopy. (a) Live HeLa cells transiently transfected with miRFP704nano N‐ and C‐terminal fusion constructs. The N‐terminal fusions are α‐tubulin, β‐actin, myosin, and vesicular protein clathrin. The C‐terminal fusions are lysosomal membrane glycoprotein LAMP1 and histone H2B. (b) Dissociated live rat cortical neurons transfected with miRFP704nano. Scale bars, 10 μm.

To evaluate the performance of miRFP704nano as a fluorescent probe for live‐cell imaging, we constructed various N‐ and C‐terminal labeled fusion proteins (Figure 7a). All fusions exhibited proper localization patterns, including fusions forming filaments, such as α‐tubulin, β‐actin, and myosin. Fusion with histone H2B localized properly in different phases of cell division.

We also expressed miRFP704nano in isolated rat cortical neurons. Notably, the primary neurons expressing miRFP704nano exhibited bright fluorescence without any supply of exogenous BV (Figure 7b).

## 3 DISCUSSION

By applying rounds of molecular evolution to the first CBCR‐based NIR FP, miRFP670nano, we have developed the second generation of miRFPnanos that efficiently binds endogenous BV and brightly fluoresces in various mammalian cells. With a molecular weight of only 17 kDa, miRFPnanos are 2‐fold smaller than BphP‐based NIR FPs and 1.5‐fold smaller than GFP‐like FPs and could be used for the tracking of small proteins for which bigger FP tags could interfere with their functions.

miRFP670nano3, miRFP704nano, and miRFP718nano are spectrally close to their two‐domain BphP‐based analogs miRFP670, miRFP703, and miRFP720 (Shcherbakova et al., 2016, 2018). The cellular brightness of miRFP704nano and miRFP718nano is comparable to that of their two‐domain analogs miRFP703 and miRFP709. On the other hand, miRFP670nano3 exhibits more than 4‐fold higher cellular brightness than that of miRFP670nano and is the brightest monomeric NIR FP developed so far.

Employing analysis of miRFP670nano3 and miRFP718nano structures we have developed new red‐shifted single‐domain NIR FP miRFP704nano. With emission maximum at 704 nm, miRFP704nano is 34 nm red‐shifted compared to miRFP670nano and miRFP670nano3, but 14 nm blue‐shifted compared to miRFP718nano (Figure 5b–d). Thus, miRFP704nano completed the spectral palette of the miRFPnano proteins series.

miRFP704nano is highly tolerant to both acidic and alkaline environments, which makes it a favorable fluorescent tag for studying protein traffic and biological processes in different cellular compartments, including those with extreme pH values (Figure 6a). miRFP704nano demonstrated substantially increased photostability, which 2.3‐fold outperformed the photostability of miRFP670nano (Figure 6b). Notably, miRFP704nano is 6.5‐ and 3.2‐fold more photostable than BphP‐based miRFP709 and miRFP703, respectively (Table 1), which makes it useful for long time‐lapse imaging. Combined high brightness, photostability, and compact single‐domain fold make miRFP718nano favorable NIR fluorescent tag for live‐cell imaging of different intracellular structures (Figure 7).

Crystal structures of miRFPnanos elucidate the observed biochemical, biophysical, and spectral characteristics of the proteins. C57 residue, introduced in miRFP704nano and miRFP718nano, binds BV via the C3^2^ atom. This binding preserves the double bond between C2 and C3 atoms and engages them in conjugation with BV, providing the red emission shift. In miRFP670nano3, on the other hand, the C86 residue binds BV via the C3^1^ atom, and the double bond is formed between C3^1^ and C3^2^ atoms and not between C2 and C3 atoms. This reduces the number of conjugated double bonds in the chromophore and causes a blue emission shift. The large hydrophobic core and compact protein structure of miRFPnanos are in good agreement with their high stability.

The available space, amount of water molecules, and the flexibility of the chromophore‐binding pocket have been proposed as the main factors to explain the spectral behavior of phytochromes (Toh et al., 2010; Wang et al., 2020; Fischer et al., 2021). Residues D207, H260, Y263, and H290 (numbering according to DrBphP) have been shown to play a role in the spectral fine‐tuning of the photoresponses (Song et al., 2013, 2011; Velazquez Escobar et al., 2017; Kirpich et al., 2018; Woitowich et al., 2018; Kraskov et al., 2021; Lenngren et al., 2018; Lehtivuori et al., 2022). The corresponding residues in CBCRs are D56, Y87, L/F90, and H117. However, in contrast to its counterpart, the D56 residue forms H‐bonds with the rings A, B, and C of the chromophore and plays the role of pyrrole water, providing for protonation/deprotonation of the phytochromes chromophore (Wagner et al., 2005). D56 of CBCRs is part of a regular β‐strand β3, whereas, in phytochromes, D207 is part of a kink between β‐strand β8 and α‐helix α6 (Wagner et al., 2005). In superimposed structures of CBCR and phytochrome (Figure 2d), it could be seen that these aspartates are rotated by nearly 180**°** relative to each other. Hence, D207 interacts with BV via its main chain carbonyl, and D56 interacts with BV via its side chain. Like H290 in DrBphPs, H117 in CBCRs forms an H‐bond with the carbonyl of the D ring, preventing its rotation around the C15=C16 bond and shifting the equilibrium towards radiative decay. As their counterparts in DrBphPs (residues H260, Y263, and H290), Y87, L/F90, and H117 control the water content in the chromophore‐binding pocket and protonation/deprotonation of the chromophore (Lenngren et al., 2018; Lehtivuori et al., 2022). In miRFPnanos, the immediate chromophore environment is enriched by hydrophobic residues and contains virtually no water molecules. Even the conserved pyrrole water found in numerous bacterial phytochromes and first described in Wagner et al. (2005) is lacking. The water content in the vicinity of the chromophore has been traditionally linked to a reduced fluorescence as hydrogen bonding with it favors vibrational relaxation pathways (Hsieh et al., 2013; Hochreiter et al., 2015; Lehtivuori et al., 2015).

Notably, Lehtivuori et al. (2022) demonstrated that adding hydrophobic residues close to the chromophore results in a brighter protein and improved pH stability. The authors performed the crystallographic and spectroscopic studies of the wild‐type chromophore‐binding domain (CBD) of *Deinococcus radiodurans* bacterial phytochrome (DrBphP) and its three mutants: CBD‐WT, CBD‐Y263F, CBD‐H260A, and CBD‐H260A/Y263F. They found that in CBD‐H260A, the removal of the histidine side chain increased the water content in the BV binding pocket, resulting in decreased quantum yield and fluorescence pH dependence. In CBD‐Y263F, the introduction of phenylalanine reduced the number of water molecules near the chromophore and increased the quantum yield of the protein. Similarly, the replacement of L90 with Phe in miRFP718nano presumably contributed to the bathochromic shift of its fluorescence.

The mutations acquired by miRFP670nano3, miRFP704nano, and miRFP718nano in the course of random mutagenesis are summarized in (Figures 2 and 5a). Four mutations in the vicinity of the chromophore deserve special consideration as they directly affect its photophysical characteristics: F90 that increased the hydrophobicity of the chromophore environment and provided for a bathochromic shift in miRFP718nano; L84 that increased the hydrophobicity of the chromophore environment in miRFP670nano3 and miRFP704nano; and M140 that reinforced the hydrophobic cluster formed by the residues L8, T/I11, V26, L/I104, and L/F114 (Figures 3f and 4f). All other mutations are located on the surface. It is highly improbable that the mutations near the chromophore are solely responsible for the drastically different photochemical properties of miRFPnanos described here. On the contrary, we believe that surface mutations act in concert with those identified above to fine‐tune the shape of the binding pocket to allow for more efficient accommodation of BV. The documented mutations could guide fine‐tuning the specific properties of this class of fluorescent biomarkers. Mutations introduced in miRFP670nano3 and miRFP704nano provided for enhanced brightness of these proteins, while mutations appeared in miRFP718nano resulted in the bathochromic shift, which frequently occurs at the expense of brightness (Matlashov et al., 2020).

NIR fluorescence makes miRFPnanos useful probes for microscopy and deep‐tissue imaging. Using two‐photon microscopy miRFP670nano3 was imaged in the somatosensory cortex of a mouse brain at the depth of up to 780 μm (Oliinyk et al., 2022). miRFP718nano was used for short‐wave infrared (SWIR) imaging in a mouse model of liver inflammation (Oliinyk et al., 2023).

With the development of miRFP704nano, the full range of CBCR‐based single‐domain 17 kDa NIR FPs has become available (miRFP670nano3, miRFP704nano, and miRFP718nano). This will allow substituting the spectrally‐similar twice‐larger two‐domain BphP‐based NIR FPs (Shcherbakova, 2021; Li et al., 2021) with these small miRFPnanos, ultimately resulting in twice smaller NIR reporters, biosensors, and protein tags for multicolor imaging at subcellular and organismal scales.

## 4 MATERIALS AND METHODS

### 4.1 Bacterial plasmids, protein expression and characterization

Genes encoding miRFP704nano and its mutants were cloned into a pBAD/His‐B vector (Thermo Fisher Scientific) by using the KpnI and EcoRI restriction sites. The oligonucleotide primers for PCR amplification and saturated mutagenesis were purchased from Biomers. Saturated mutagenesis or site‐specific mutagenesis was performed using overlap‐extension PCR. FACS screening of mutant libraries was performed with an Influx cell sorter (BD Biosciences), using a 640 nm excitation laser and 725/40 nm emission filter. Screening of the brightest clones was performed with a Leica M205 fluorescence stereomicroscope equipped with a CCD camera (Tucsen), using 700/20 nm excitation and 730 nm long‐pass emission filters (Chroma). Around 30 brightest red‐shifted clones were subcloned into a pcDNA3.1 plasmid (Invitrogen/Thermo Fisher Scientific) and tested in transiently transfected HeLa cells.

Proteins were expressed in LMG194 bacterial cells, co‐transformed with pWA23h plasmid, encoding heme oxygenase. Bacterial cells were grown to an optical density of 0.5–0.7 at 600 nm in LB/ampicillin/kanamycin medium supplemented with 0.02% rhamnose and, then, to induce miRFPnano expression, 0.005% arabinose was added. Bacteria were cultured for 5 h at 37°C and then, at 22°C for 20 h. Protein purification was performed with Ni‐NTA agarose followed by size‐exclusion chromatography. The Ni‐NTA agarose column (Macherey‐Nagel) was equilibrated with a buffer containing 50 mM NaH_2_PO_4_ and 300 mM NaCl at pH 8.0. For the washing step, 20 bed volumes of the equilibration buffer containing 10 mM imidazole were used. For the protein elution, the equilibration buffer containing 100 mM EDTA was used. The size‐exclusion liquid chromatography was performed using the HiLoad 16/600 Superdex‐200 column (GE Healthcare). The column was equilibrated with a buffer containing 10 mM HEPES, 150 mM NaCl, 10% glycerol, 50 μM EDTA, 1 mM dithiothreitol, 0.2 mM phenylmethylsulfonyl fluoride, 0.01% EP‐40, and 0.2 mM benzodiazepine (pH 7.4). The flow rate of 1 mL/min was used.

Fluorescence spectra were recorded with a Cary Eclipse fluorimeter (Agilent Technologies). Absorbance measurements were performed with a Hitachi U‐2000 spectrophotometer.

The extinction coefficient of miRFP704nano was determined as a ratio between the absorbance value of the peak at the Q‐band and the value of the peak at the Soret band, given a Soret band extinction coefficient of 39,900 M^−1^ cm^−1^ (Filonov et al., 2011). The fluorescence quantum yield of miRFP704nano was determined using miRFP709 as a standard. A ratio of the integrated fluorescence intensities of the miRFP704nano and miRFP709 samples at several dilutions with identical absorbance was assumed as the ratio of the quantum yield values. pH stability was studied using a series of Hydrion buffers (Micro Essential Laboratory). Data fitting and statistical analysis were performed using OriginPro 2021b v.9.8.5.212 (OriginLab) and Excel v.15.36 (Microsoft) software.

### 4.2 Protein crystallization

For crystallization, miRFP670nano3 was equilibrated in 20 mM Tris–HCl and 300 mM NaCl at pH 8.0 buffer and concentrated to 27.3 mg mL^−1^. Initial crystallization conditions were found with the NT8 crystallization robot (Formulatrix) using Hampton Research, Jena Bioscience, and Molecular Dimensions screens. The conditions were further optimized with additive screens. The best crystals could be obtained from 8.4% PEG 4000, 3.6% MPD, 0.06 M sodium/potassium phosphate buffer pH 6.3. The crystals suitable for X‐ray data collection were grown by the hanging‐drop vapor diffusion method. In the large‐scale crystallization experiment, 2 μL of the protein solution was mixed with 2 μL of the reservoir solution and incubated against 500 mL of the same reservoir solution at 20°C for a week.

### 4.3 Determination of protein structures

X‐ray data were acquired on the SER‐CAT 22‐BM beamline station (Advanced Photon Source, Argonne National Laboratory, Argonne, IL). Before data collection, the crystals were flash‐frozen in a 100 K nitrogen gas stream. Diffraction images were processed with HKL2000 (Otwinowski and Minor, 1997). The structure of miRFP670nano3 was solved by the molecular replacement method with MOLREP (Vagin and Teplyakov, 1997) using the structure of miRFP670nano (PDB ID: 6MGH) as a search model. To remove model bias, the structure was rebuilt with ARP/wARP model building and density improvement software (Lamzin et al., 2012). The structure refinement was carried out with REFMAC5 (Murshudov et al., 2011) (CCP4 suite) and PHENIX.REFINE (Adams et al., 2010) (PHENIX suite) programs. Real‐space model correction and structure validation were performed with COOT (Emsley et al., 2010). The data collection and refinement statistics are given in Tables 2 and 3, respectively.

**TABLE 2:** Data collection statistics for miRFP670nano3.

Space group	*C*2
Unit cell parameters (Å, °)	*a* = 111.5
	*b* = 74.0
	*c* = 83.7
	*β* = 101.7
Temperature (K)	100
Wavelength (Å)	1.00
Resolution (Å)	30–1.8
Total reflections	274,220
Unique reflections	60,575
Completeness (%)	98.5 (93.6)
*I*/*σ*<*I*>	16.1 (1.7)
R‐merge	0.074 (0.82)
Multiplicity	4.5 (3.9)

*Note*: Data in parentheses are given for the outermost resolution shells 1.86–1.8 Å.

**TABLE 3:** Refinement statistics miRFP670nano3.

No. of protein atoms	5120
No. of solvent atoms	371
Resolution range (Å)	30–1.8
R‐work	0.178
R‐free	0.237
RMSD bond lengths (Å)	0.010
RMSD angles (°)	2.28
RMSD chirality (°)	0.096
RMSD planarity (°)	0.011
RMSD dihedral (°)	19.5
Mean B factors (Å^2^)	
Protein atoms	
Overall	40.0
Main chain	36.6
Side chain	43.2
Chromophore	79.3
Water	47.2
Ramachandran statistics (%) (for non‐Gly/Pro residues)	
Most favorable	96.0
Additional allowed	3.4
Generously allowed	0.6

### 4.4 Construction of mammalian plasmids

To construct plasmids encoding miRFPnanos, the respective genes were inserted into the pcDNA3.1 plasmid (Invitrogen/Thermo Fisher Scientific) at KpnI/EcoRI sites. To engineer plasmids for labeling of intracellular structures, the gene encoding miRFP704nano was swapped with the gene encoding miRFP670nano in the plasmids pTubulin‐miRFP670nano (Addgene #127429), pActin‐miRFP670nano (Addgene #127428), pMyosin‐miRFP670nano (Addgene #127431), pClathrin‐miRFP670nano (Addgene #127430), pLAMP1‐miRFP670nano (Addgene #127435), and pH2B‐miRFP670nano (Addgene #127438).

### 4.5 Mammalian plasmids, cell culture and transfection

HeLa (CCL‐2), N2A (CCL‐131), U‐2 OS (HTB‐96), HEK293T (CRL‐3216), and NIH3T3 (CRL‐1658) cells were obtained from the ATCC. Cells were cultured in a DMEM medium supplemented with 10% FBS, 0.5% penicillin–streptomycin and 2 mM glutamine (Invitrogen/Thermo Fisher Scientific) at 37°C. For live‐cell fluorescence microscopy, cells were plated in 35 mm glass‐bottom Petri dishes (Greiner Bio‐One International). Transient transfections were performed using polyethyleneimine (Moutel et al., 2016) or Effectene reagent (Qiagen).

### 4.6 Neuronal culture and transfection

Rat cortical neurons were prepared in the Neuronal Cell Culture Unit of the University of Helsinki. All animal work was performed under the ethical guidelines of the European Convention and regulations of the Ethics Committee for Animal Research of the University of Helsinki. The embryos staged at E17–E18 from the female rats were used. Animals were kept in standard conditions. Neurons were plated at a density of 500,000–700,000 per 35 mm glass‐bottom dish, coated with poly‐l‐lysine (0.01 mg mL^−1^) (Merck). Neurons were grown at 37°C and 5% CO_2_ in a neurobasal medium (Gibco) supplemented with B27 (Invitrogen/Thermo Fisher Scientific), l‐glutamine (Invitrogen/Thermo Fisher Scientific) and penicillin–streptomycin (Lonza). Cultured neurons were transfected with pcDNA plasmids encoding miRFP704nano at 4–5 days in vitro (DIV) using Effectene Transfection Reagent (Qiagen) and imaged 48 h after transfection.

### 4.7 Studies of intracellular protein stability

For the protein degradation assay, HeLa cells were transiently transfected with plasmids encoding miRFP704nano, miRFP718nano, miRFP670nano and EGFP. 48 h after transfection cells were incubated for 4 h with 10 μM bortezomib or cycloheximide (20 μg mL^−1^), respectively. Then, the fluorescence of control non‐treated cells, cells treated with bortezomib and cells treated with cycloheximide was analyzed by flow cytometry.

To analyze the brightness of fluorescent cells at different time points after transfection, HeLa cells were transiently transfected with miRFP704nano‐, miRFP718nano‐, or miRFP670nano‐ and EGFP‐encoding plasmids. The cells were passaged on day 2 after transfection. The fluorescence intensities were analyzed by flow cytometry on days 2 and 5 after transfection.

### 4.8 Mammalian cell imaging

Live cells were imaged with an Olympus IX81 inverted epifluorescence microscope, equipped with a Xenon lamp (Lambda LS, Sutter). An ORCA‐Flash4.0V3 camera (Hamamatsu) was used for image acquisition. Cells were imaged using a 60× 1.35 NA oil objective lens (UPlanSApo, Olympus). During imaging, HeLa cells were incubated in a cell imaging solution (Life Technologies‐Invitrogen) and kept at 37°C. The microscope was operated with a SlideBook v.6.0.8 software (Intelligent Imaging Innovations). To image miRFP704nano a 685/20 nm exciter and a 725/40 nm emitter (Chroma) were used.

Photobleaching measurements were performed in live HeLa cells 48 h after the transfection using a 60× 1.35 NA oil objective lens (UPlanSApo, Olympus). Obtained raw data were normalized to corresponding absorbance spectra and extinction coefficients of the miRFPnanos, the spectrum of the Xenon lamp and the transmission of the 665/45 nm excitation filter.

The data were analyzed using SlideBook v. 6.0.8 (Intelligent Imaging Innovations) and Fiji v.1.50b software.

## ACCESSION CODE

The crystal structure of miRFP670nano3 was deposited with Protein Data Bank under accession code 7LSC.

## AUTHOR CONTRIBUTIONS

Olena S. Oliinyk developed the miRFP704nano protein and characterized it spectrally, biochemically and in cells. Sergei Pletnev designed the structural biology experiments and analyzed the data. Mikhail Baloban expressed and purified the recombinant proteins. Vladislav V. Verkhusha planned and supervised the whole project, discussed the results and planned the experiments. All authors wrote the manuscript.

## CONFLICT OF INTEREST STATEMENT

The authors declare no competing interests.
